# Fluorescence hyperspectral imaging (fHSI) using a spectrally resolved detector array

**DOI:** 10.1002/jbio.201600304

**Published:** 2017-05-09

**Authors:** Anna Siri Luthman, Sebastian Dumitru, Isabel Quiros‐Gonzalez, James Joseph, Sarah E Bohndiek

**Affiliations:** ^1^ Department of Physics University of Cambridge JJ Thomson Avenue Cambridge CB3 0HE U.K.; ^2^ Cancer Research UK Cambridge Institute University of Cambridge, Li Ka Shing Centre Robinson Way Cambridge CB2 0RE U.K.

**Keywords:** multiplexed fluorescence, biomedicial, hyperspectral, SRDA, *in vivo*, instrumentation

## Abstract

The ability to resolve multiple fluorescent emissions from different biological targets in video rate applications, such as endoscopy and intraoperative imaging, has traditionally been limited by the use of filter‐based imaging systems. Hyperspectral imaging (HSI) facilitates the detection of both spatial and spectral information in a single data acquisition, however, instrumentation for HSI is typically complex, bulky and expensive. We sought to overcome these limitations using a novel robust and low cost HSI camera based on a spectrally resolved detector array (SRDA). We integrated this HSI camera into a wide‐field reflectance‐based imaging system operating in the near‐infrared range to assess the suitability for *in vivo* imaging of exogenous fluorescent contrast agents. Using this fluorescence HSI (fHSI) system, we were able to accurately resolve the presence and concentration of at least 7 fluorescent dyes in solution. We also demonstrate high spectral unmixing precision, signal linearity with dye concentration and at depth in tissue mimicking phantoms, and delineate 4 fluorescent dyes *in vivo*. Our approach, including statistical background removal, could be directly generalised to broader spectral ranges, for example, to resolve tissue reflectance or autofluorescence and in future be tailored to video rate applications requiring snapshot HSI data acquisition.

## 1 Introduction

HyperSpectral Imaging (HSI) is a powerful analytical tool based on the detection of both spatial and spectral information within a single data set, referred to as a HSI cube (x‐y‐λ). Originally conceived for remote sensing 1, HSI has impacted fields as diverse as food inspection 2 and forensics 3. The power of HSI lies in the ability to determine the chemical composition of a sample based on characteristic spectral signatures, using multivariate statistical methods to produce a spatial map of the chemical constituents.

The ability to detect multiple fluorescent labels targeted to different processes is highly advantageous across a range of length scales in biomedical imaging: from studying complex molecular interactions in cells at super‐resolution 4; through monitoring contrast agent biodistribution in small animals 5, 6; to diagnosing, characterising and resecting cancer in humans 7, 8. Biomedical application of HSI has already enabled numerous fluorescent contrast sources (e. g. small molecule dyes, fluorescent proteins, autofluorescence) to be delineated within a single data set. Implementations of fluorescence HSI (fHSI) systems in microscopic 9 and small animal imaging 10, 11 based on tunable spectral filters have gained commercial traction 12, 13, however, in practice these systems are typically used to resolve fewer than 3 fluorescent dyes or proteins 9, 14, 15, 16, 17. Increasing the number of resolvable labels to *>*5 has required the use of narrowband emitters such as quantum dots 18 or specialized sample geometry 19.

**Figure 1:**
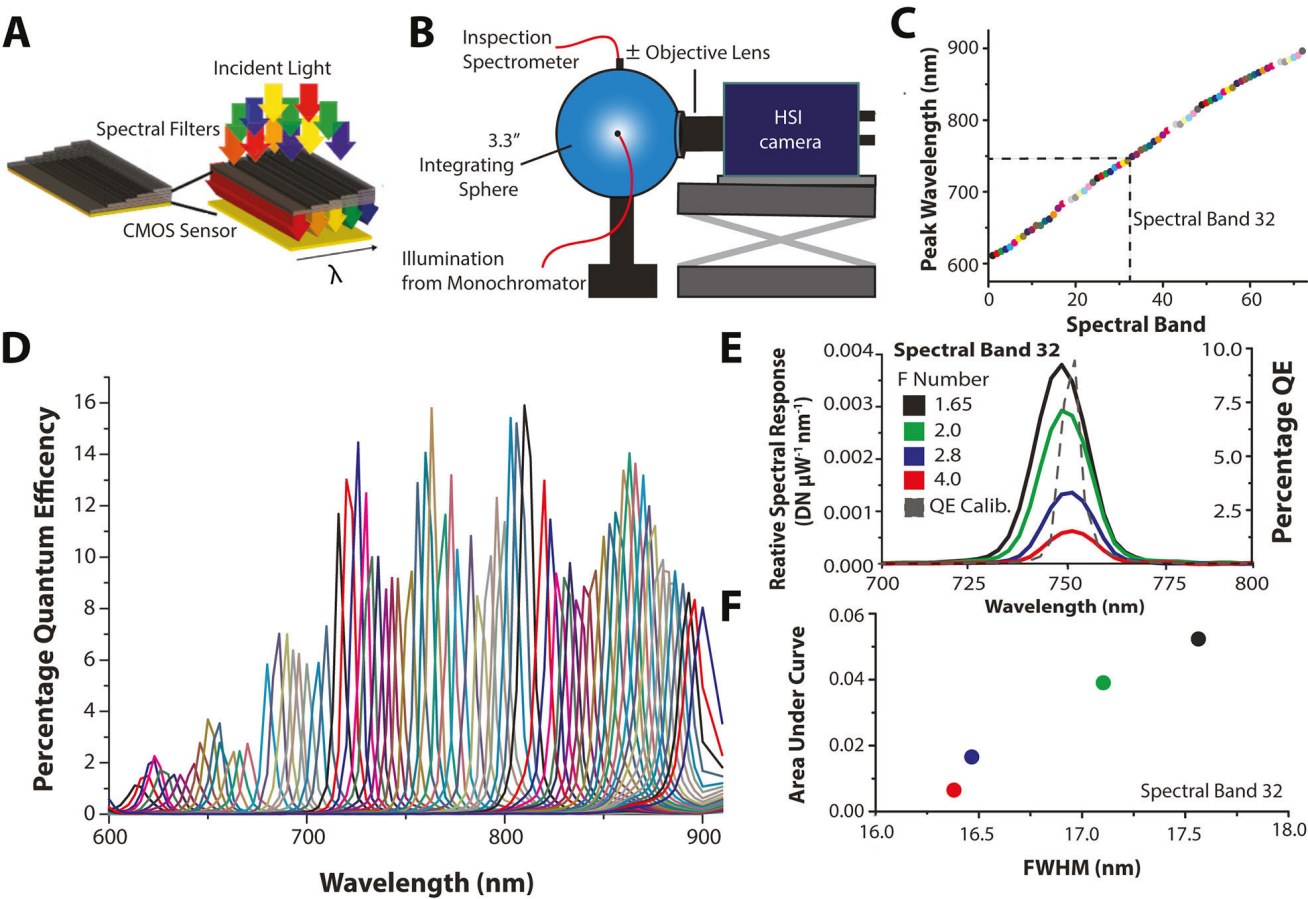
Characterization of the hyperspectral imaging (HSI) camera. (A) The HSI camera contains a CMOS sensor with row‐wise deposition of Fabry‐Pérot etalon cavities (simplified schematic); each ‘spectral band’ occupies 8 pixel rows. (B) Experimental setup for calibration. (C) The calibration procedure located 72 spectral bands with peak wavelength between 600 and 900 nm; here shown for a camera objective F/# of 1.65. (D) The quantum efficiency as a function of wavelength – QE(λ) – for each of the 72 spectral bands. As the F/# of the coupled camera objective increases, the total signal falling on the sensor decreases (E), whilst the full width at half maximum (FWHM) narrows (F) differing notably from the QE(λ) collimated light (F, dashed line). Data in (E) and (F) are taken from spectral band 32.

To date, translating the power of fHSI into applications in humans, such as endoscopic and intra‐operative imaging, has been challenging due to the need for complex, bulky and expensive instrumentation with limited frame rate. Nonetheless, the ability to characterize both endogenous tissue contrast based on diffuse reflectance and to identify the presence and concentration of exogenous small molecule fluorescent dyes 17, 20 is highly desirable in the clinic.

Spectrally resolved detector arrays (SRDAs) – where the spectral filters are deposited on a glass substrate either in front of 21, 22, or directly on top of, the sensor array 23, 24 – are promising for future clinical application of fHSI. SRDAs are extremely compact and robust and therefore well suited for use in an endoscopy suite or operating theatre. They also have the potential to be produced at very low cost, as the filters may be deposited on the sensor array during the lithography process 23. When the spectral filters are deposited on the sensor in a pixel‐wise mosaic pattern, video rate spectral imaging is possible 24. Encouragingly, early applications of snapshot SRDA have shown promise in neurosurgery 25 and ophthalmology 26 using white‐light reflectance imaging.

Here, we evaluated the performance of an SRDA‐based HSI camera for fluorescence imaging, with a view to future clinical application. While the SRDA filters can be deposited pixel‐wise for real‐time ‘snapshot’ imaging 24, 25, 26, we used row‐wise filters to perform ‘pushbroom’ HSI in order to maintain high spatial and spectral resolution in this initial technology evaluation. The utility of the ‘pushbroom’ SRDA used in this study has been explored in a wide range of applications, from food inspection to agricultural monitoring 27. Following detailed characterization of the HSI camera, we created a reflectance‐based wide‐field imaging system in order to provide a first proof‐of‐concept for fHSI using an SRDA. We evaluated: HSI response linearity; spectral unmixing precision; and delineation of reflectance and fluorescence light in tissue. The wide‐field fHSI system based on our SRDA can resolve at least 7 fluorescent dyes in solution and at least 4 in small animals, showing promising performance for future clinical application.

## 2 Methods

### 2.1 Hyperspectral imaging camera and calibration

The HSI camera SRDA (Imec) monolithically integrates a set of 100 Fabry‐Pérot etalon filters between 600 and 1000 nm (Figure 1A) on a CMOS sensor (CMV2000; CMOSIS) packaged within a camera system (Q‐2 A340 m/CL; Adimec). The Fabry‐Pérot etalon filters are row‐wise deposited on the sensor to form spectral bands; each spectral band occupies 8 pixel rows. Transmission efficiencies of up to 73 % have been reported for the filters in this HSI camera SRDA 23. For all measurements, integration time was set to be 99.73 ms, frame period to 100 ms, gain to 1 and 2 tap acquisition of 10 bit images (without image averaging) in linear mode (dynamic range of 60 dB) was used. Spatial calibration of filter row position was achieved by identifying the pixel rows that responded similarly to diffuse light at each wavelength based on a city block metric analysis 28. We measured photon transfer curves 29 with the HSI camera by illuminating the sensor directly using two broadband LED arrays (Aspect Systems) with peak emission wavelengths of 635 nm and 850 nm respectively. The conversation gain was found to be 0.16±0.01 e‐/DN and the readout noise was found to be 95±0.07 DN (mean ± standard error across spectral bands). HSI camera quantum efficiency (including optical filter transmission and sensor response) as a function of illumination wavelength was measured using a commercial calibration system (Aspect Systems) according to standard methods 29, 30 with collimated light produced using a bi‐convex lens (LB1723‐B; Thorlabs). Quantum efficiency (QE) was defined as
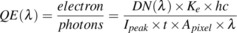



where DN(λ) is the digital number of the averaged, dark subtracted pixels within the spectral bands, *Ke* is the conversion gain of the sensor, *I_peak_* the peak photon intensity measured with the calibrated photodetector, *t* the integration time and *A_pixel_* the pixel area 30. Imaging was performed using an objective lens (35 mm VIS‐NIR Compact Fixed Focal Length Lens, Edmund Optics) and 600 nm IR long pass filter (Imec). To assess the impact of illumination angle (F/#), we used a custom monochromator‐integrating sphere system (Figure 1B). A broadband halogen light source (OSL2 with OSL2B2 bulb; Thorlabs) was coupled via a 0.48 NA 1000 μm diameter fiber (M71 L01; Thorlabs) into a monochromator (CM110 1/8 m; Spectral Products). The monochromatic light was then relayed via an optical fiber (M71 L01; Thorlabs) to an integrating sphere (AS‐02994‐033; Labsphere). Isotropically diffuse light output from the integrating sphere was directly input to the camera objective at the 1.5” sphere output port. The focal length of the objective was kept at infinity throughout the measurements to avoid focusing on the internal structure of the integrating sphere. The monochromator was scanned between 580 and 920 nm in 3 nm increments and intensity spectra were acquired via a calibrated inspection spectrometer (AvaSpecULS2048‐USB2‐FCDC; Avantes). We defined the ‘relative spectral response’ recorded with the objective lens in place as
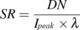



where SR is the baseline corrected relative spectral response of the camera in DNμW^−1^nm^−1^, DN is the digital number output of the camera, λ the peak wavelength output from the monochromator and *I_peak_* is the peak intensity recorded by the inspection spectrometer in μWcm^−2^.

### 2.2 Wide field fHSI system design

The HSI camera was mounted onto a strong aluminium frame (XT95‐500, XT95P3, XT95P12/M and XT95P11/M; Thorlabs) above the sample using a custom adapter, designed to minimize mechanical vibrations. The system was enclosed in a light tight box. Sample translation for line scan imaging was achieved using a motorized translation stage (LTS150; Thorlabs), which was synchronized to the process of image acquisition. The sample was consecutively translated a distance equivalent to that of one spectral band before each image acquisition. Our alignment procedure has been detailed elsewhere 28. Briefly, alignment was achieved by acquiring an HSI cube from a printed grid, placed on top of a reflectance target (Lambertian White Screen, SG3151‐0; Sphere Optics) in the sample plane and illuminated with a halogen lamp. Fine tuning of sample positioning orthogonal to the scanning was achieved using manual stages (L200/M, PT1/M; Thorlabs). The height of the sample stage and the focus of the objective were adjusted such that the field‐of‐view (FOV) was 70 mm×20 mm; the smallest FOV was used to maximize the detected fluorescence signal. For an objective F/# of 1.65, the spatial resolution at this FOV was determined to be 0.56 lines/mm using a USAF test chart (R1DS1N; Thorlabs). No noticeable keystone or spectral smile aberrations were observed in the image data (see Supporting Figures S1 and S2).

Sample illumination was provided by a custom LED ring of 50 mm internal radius. Three colors of high power LEDs with peak wavelengths 590, 660, and 732 nm (LZ1‐10 A100, LZ1‐10R200, LZ1‐10R30; MCPCB mounted from LED Engine) were mounted on a copper ring, each LED controlled via a separate current controller (LEDD1B; Thorlabs) with negligible intensity drift (*<*2 %). Six LEDs of each type were connected in series equidistant around the internal rim of the ring and photographic diffuser paper (Lee White Diffusion 129; Calumet Photographic) was placed underneath. We compared data acquired with and without crossed linear polarisers (LPVISE2X2; Thorlabs) to compare hardware specular reflection removal with our background removal methods implemented in software.

The power density at the sample was calibrated using an optical power meter (1916‐R; Newport) centrally placed in the sample area. Spatial illumination uniformity was mapped separately for each LED color at 100 mW/cm^−2^, with and without the crossed linear polarisers, by translating an optical power meter in 0.5 cm steps in x and y across the sample area. The spatial non‐uniformity did not exceed 5 % for the LEDs without crossed polarisers. The addition of crossed polarisers (and removal of the diffusion paper) substantially affected the uniformity of the illumination increasing to 29 % at 660 nm due to a combination of the removal of the diffusion paper and the low quality of the polarizing filters.

### 2.3 Data Analysis

For every experiment, a dark and ‘flat’ HSI cube were acquired respectively by turning off all LEDs or by illuminating the sample prior to addition of the fluorescent dyes. Raw data is available by contacting the corresponding author of the manuscript. Initial pre‐processing steps (see Supporting Methods for more details) include: dark subtraction, to account for any sensor non‐uniformities; edge correction, to account for a slight mismatch with the filter edges and pixel positions (Supporting Figure S3); and spatial vignetting correction, using an HSI cube acquired under broadband illumination in the sample plane to provide a vignetting correction mask for each spectral band. Finally, background removal is performed to address the presence of specular reflections in our data, which comprise 1–5 % of the image pixels in well plate and tissue phantom data. Strong specular reflections in the well plates arise due to meniscus effects in the droplets containing fluorescent dyes, and moist or wet areas in the tissue phantoms. For mouse skin, which is more diffusely reflective, less than 0.003 % of image pixels are affected. A ratiometric thresholding method was developed to remove areas of strong specular reflection from our quantitative analysis (see Supporting Methods).

We tested two software background removal methods, which exploit the spectral dimension of the information collected in the HSI cube, and compared the result to the inclusion of crossed linear polarisers in the hardware, which remove only surface specular reflections. The advantage of performing background removal in software, assuming we are able to achieve a comparable performance for reflectance removal as in hardware, is the potential to extract information from the diffuse reflectance light in a clinical scenario. The ‘brute force’ software approach mimics the action of applying optical band‐pass filters to block the reflectance light. The maximum signal intensity of the reflected LED illumination for the power levels chosen in this study was first determined by imaging the uniform reflectance target under our standard illumination conditions. For spectral bands 9–17 and 21–35, which would be adversely affected by reflectance of the LED illumination, bands where the signal intensity exceeded 10 % of the measured maximum reflectance intensity were then rejected from the analysis. The spectral bands rejected were kept fixed throughout the study. Orthogonal subspace projection (OSP) 34 uses the ‘flat’ HSI cube to obtain the principal components of the background. A matrix of orthogonal basis spectra V was taken from the background HSI cube (5 most significant principal components). The projections of V on the original endmembers (*a*) were then subtracted to produce background removed endmembers (*r*, input to spectral unmixing as described below) according to *r*=*a* −*aV* ^*T*^ *V* . This approach has the advantage of removing not only specular reflections but also any other sources of stray background light.

‘Endmembers’ are the spectra to be fitted using spectral unmixing. To account for system‐dependent variations in the recorded spectral profiles, we used the spectra of the fluorescent dyes recorded by the wide field fHSI system in a single reference measurement 14, 16 of the fluorescent dyes at 40 μM concentration in a well plate. Endmembers were extracted as average spectra from manually placed ROIs (20 pixels radius) positioned at the known position of the dye in the pre‐processed ‘reference’ HSI cube. The background endmember was obtained by averaging the pixel spectra from the pre‐processed ‘flat’ HSI cubes. Separate dye dilutions were prepared for the acquisition of each ‘reference’ HSI cube, meaning that endmembers were always collected from a separate sample than that being classified. Classification was achieved by non‐negativity constrained least squares (NNLS) spectral unmixing using the Matlab function lsqnonneg. The result of the spectral unmixing procedure is an abundance map for each of the fluorescent dyes and one for the background. ROIs were placed over the abundance maps; for the well plate data a ratiometric thresholding method was used to avoid areas of strong specular reflections (see Supplementary Informations for further details).

### 2.4 fHSI imaging of multiple fluorescent dyes in well plates and phantoms

A 40 μM dilution of each of AF610, AF647, AF700 and AF750 (A30050, A20006, A20110 and A37568, all NHS ester; Invitrogen) was prepared in phosphate buffered saline (PBS) (10010015; Thermo Fisher). A well plate (18 well, μ‐slide, 81826; ibidi) containing 30 μL of each dye in a separate well, along with a PBS control was used to acquire the ‘reference’ HSI cube (with and without crossed polarizers), from which endmembers could be extracted. A ‘flat’ HSI cube of a well plate containing only PBS was recorded for OSP background removal. Plates containing both dilution series of each dye (1:2 steps from 10 μM to 625 nM) and mixtures of different dyes were then imaged for performance evaluation. Two duplicates of each concentration and two PBS blanks were pipetted in opposite directions along two rows of the well plate, to ensure repeatability within the FOV. Absorption and fluorescence spectra, as well as the accuracy of the dilutions, were verified using microplate readers (FLUOstar and CLARIOstar; BMG LABTECH). A further well plate was imaged containing 40 μM dilutions of three additional fluorescent dyes to the Alexa Fluors: Cyanine 7.0; Sulfo‐Cyanine 7.0; and Cyanine 7.5 (Cy7, S−Cy7 and Cy7.5, 25090, 25390, 26090; Lumiprobe). 20 μL of 40 μM dye dilutions prepared in ethanol –to avoid aggregation of the cyanine dyes – were imaged.

For phantom experiments, 1.5 % agar solution (05039‐500G; Fluka) was mixed with intralipid emulsion (20 % emulsion, I141‐100ML; Sigma‐Aldrich) to provide a scattering coefficient of 5 cm^−1^ at 633 nm. A 0.75 % volume of 0.5 mgml^−1^ nigrosin (198285‐25G; Sigma‐Aldrich) was then added to provide an absorption coefficient of 0.05 cm^−1^ at 633 nm. While liquid, the phantom base material was poured into multiple petri dishes (664160; Greiner Bio‐One) to depths of either 0.2 or 1 cm. Fluorescent target inclusions were made using transparent plastic straws with an internal diameter of 0.3 cm (391SIPCL; Plastico). The transparent straws were cut into 1 cm long pieces and one end of each piece sealed with a glue gun (PA6‐GF30: Type PXP 06; Henkel Pattex Supermatic). Eight 1 cm long straw pieces were inserted into a 1 cm thick phantom slab, with the glue sealed end directed towards the bottom of the petri dish; half of the straw inclusions were filled (50 μL) with a 40 μM solution of each of the fluorescent dyes (AF610, AF647, AF700 and AF750) in equal part phantom base material and PBS and half were filled with PBS and phantom base material. A second equivalent phantom was prepared with all the straw inclusions filled with the PBS and phantom base material mixture for the ‘flat’ HSI cube acquisition.

### 2.5 *In vivo* imaging

All animal procedures were conducted under a project license issued under the United Kingdom Animals (Scientific Procedures) Act, 1986 (70‐8214). The project license was reviewed by the local Animal Welfare and Ethical Review Board and the specific experimental protocol was approved locally by the Named Animal Care and Welfare Officer at the Cancer Research UK Cambridge Institute under compliance form number CFSB0964 and conducted by individuals holding personal licenses. 40 μM dilutions of each of the AlexaFluor dyes and an equal parts mixture of all four (from 30 μM dilutions) were prepared in a 1:1 solution of PBS and phenol‐red‐free matrigel (356231; Corning). Experiments were performed in nude mice (n=2) maintained under inhaled anaesthesia using isoflurane mixed with 100 % oxygen for a maximum of 2 hours for the entire study. During imaging on the fHSI system, the mouse was placed on a heat pad (Physiosuite Monitoring Module, Kent Scientific Co.) within a bespoke 3‐D printed animal holder. 30 μL of each dye preparation was injected subcutaneously using a Hamilton syringe into the flank of the mouse. The contralateral side of the mouse was used as an imaging control for acquisition of the ‘flat’ HSI cube. Images were also acquired using an IVIS200 (Perkin Elmer) per standard operating procedures to verify successful injection and placement of the fluorescent dyes using a filter‐based fluorescence imaging system.

## 3 Results

### 3.1 Calibration of the HSI camera

We first established the performance capabilities of our HSI camera. We selected a near‐infrared camera to be compatible with *in vivo* imaging of exogenous fluorescent dyes. The monolithically integrated spectral filters are formed from Fabry‐Pérot etalon cavities deposited over 8 pixel rows on the sensor (referred to as a ‘spectral band’, Figure 1A). The cavity size defines the center wavelength of the filter. The reflectivity of the cavity mirrors and the angle of incidence of the illumination light determine the full width at half maximum (FWHM) of the spectral response 31. To fully understand this behaviour, we used a monochromator‐integrating sphere system (Figure 1B) to characterize the response of the 72 spectral bands in the wavelength range of interest for this study (600 – 900 nm, Figure 1C). The peak QE of the spectral bands was found to vary between 1.5 % to 15.9 % when using collimated light (Figure 1D). The mean intraband standard deviation of the peak wavelength and FWHM of the spectral response of 8 pixel rows composing a spectral bands were 2.00 nm and 0.37 nm respectively. In agreement with theory 31, 32, the spectral response is highly dependent on the F/# of the objective lens used for imaging (Figure 1E,  F), with a lower optical throughput and narrower FWHM for increasing F/#. As the F/# of the objective lens is changed from 1.65 to 4.0, the optical throughput (area‐under‐curve) decreases by 84±1 %, while the FWHM decreases by 8±2 % (mean ± standard deviation across all spectral bands). The full spectral data is shown in Supporting Information Figure S4. The center wavelength also converges on the value measured using collimated light during the QE calibration. For fluorescence imaging, with low light levels and broad spectral profiles, we selected a low F/# to maximize optical throughput (set to 1.65 for the remainder of this work).

### 3.2 Design and characterization of the fluorescence HSI wide‐field system

**Figure 2:**
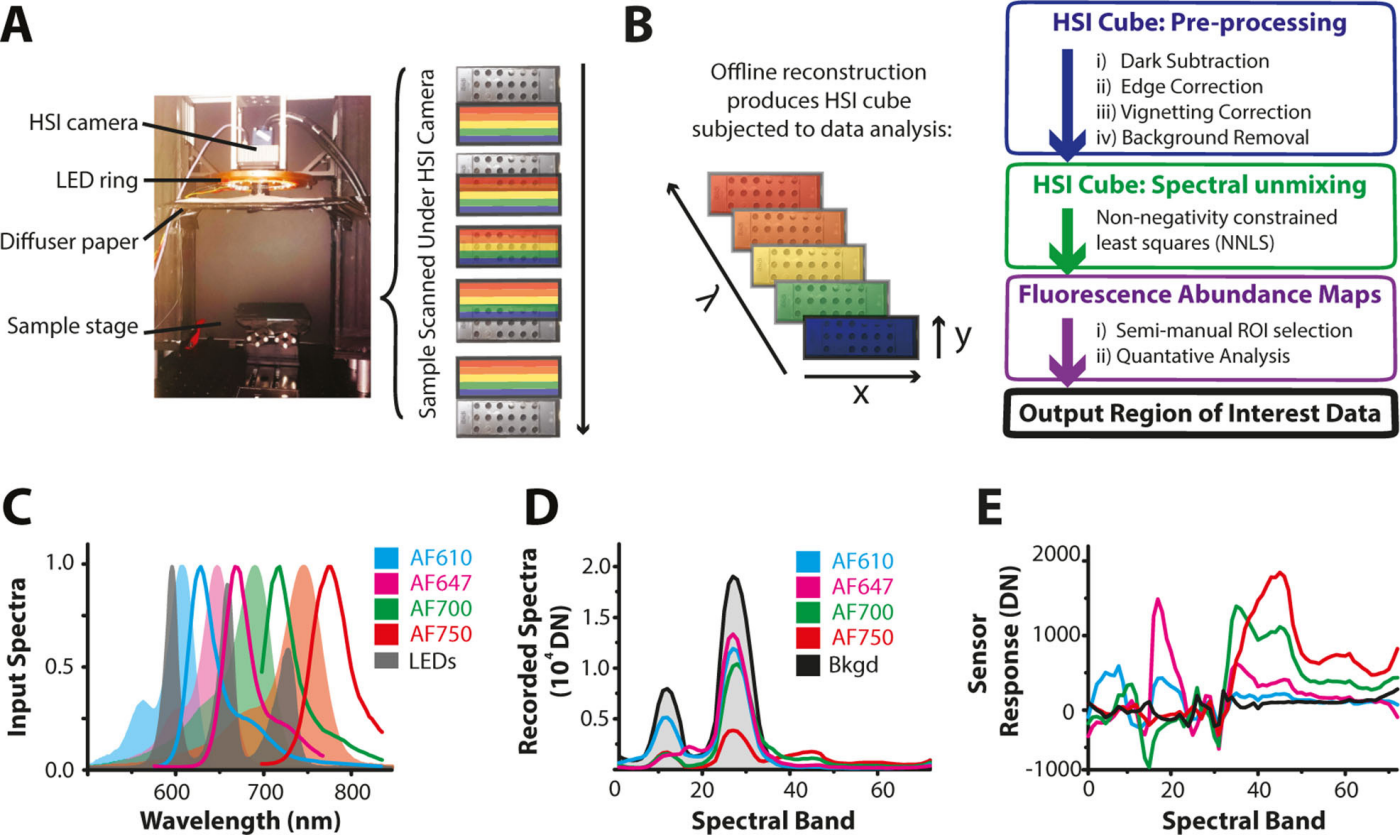
Overview of the fluorescence HSI (fHSI) wide field system. (A) The sample is scanned under the HSI camera. (B) The HSI cube is reconstructed offline by stitching together the spectral band data from each scan position. The raw HSI cube then undergoes several pre‐processing steps – including background removal prior to spectral unmixing and quantitative analysis. (C) Emission spectra of the 3 LED colors (shaded grey) overlapped with the excitation (shaded curves) and emission (line) spectra of the fluorescent dyes. Raw spectra recorded by the fHSI system for pure dye solutions (D) are used to extract endmembers, shown after background removal via orthogonal subspace projection (E).

To provide an initial proof‐of‐concept for fHSI relevant to clinical applications, we decided to create a simple reflectance‐based demonstrator system, which mimics typical sample and illumination geometry. This allowed us to specifically capture and explore the hardware and software challenges of performing fHSI without adding all of the complexities that would be associated with a fully functional endoscopic or intraoperative system. We mounted the HSI camera above a motorized translation stage (Figure 2A). The sample is scanned under the HSI camera, with a single image acquired by the HSI camera at each scan position. Each image from the HSI camera has dimensions of *x* − λ, while the line scan adds the *y* dimension. The HSI ‘data cube’ is reconstructed offline (procedure outlined in Figure 2B) after the spectral scan by stitching together the spectral data from each *y* step to produce an *x* −*y* −λ matrix (2048×576×72=84.9 MegaPixels; x‐y pixel dimension 34.2 μm in the FOV). Line scanning requires an acquisition time of up 4 minutes per HSI cube, but allows us to maximize the spectral and spatial information available in our SRDA. While acceptable in the present system, we would need to trade off spectral and spatial resolution to transition into a pixelwise filter deposition for future clinical implementations.

HSI traditionally uses either broadband white light or narrowband filters for illumination 20. We took an intermediate approach, using an light emitting diode (LED) ring composed of 3 LED colors: one outside of the spectral response range of the HSI camera at 590 nm, and two within the range at 660 nm and 732 nm (Figure 2C). LEDs were chosen to allow flexible ‘plug and play’ exchange of excitation wavelengths. We set the power density of each color to 100 μWcm^−2^ in the sample plane for all experiments. Light from all LEDs simultaneously illuminated the full FOV throughout the data acquisition.

### 3.3 fHSI of multiple fluorescent dyes in well plates

Having implemented and calibrated the system hardware, we then sought to evaluate the imaging performance in a well‐controlled sample. Four fluorescent dyes initially used to assess fHSI performance were AlexaFluor (AF) 610, 647, 700 and 750. The dyes were chosen to have a relatively high spectral peak separation (∼50 nm) and a range of quantum yields, with variable overlap between their fluorescence emissions and the reflectance of the LED excitation light (AF647 and AF700: strongly overlapping; AF610: moderately overlapping; AF750: separated; Figure 2C). We used a simple well plate with 30 μL microwells containing first each pure fluorescent dye in a 1:2 dilution series starting from a concentration of 10 μM then mixtures of the four dyes at a final concentration of 10 μM. A ‘flat’ HSI cube was acquired from a reference well plate without fluorescent dyes; an ‘endmember’ HSI cube was acquired from another plate containing only pure fluorescent dye solutions at 40 μM (Figure 2) 14, 16.

#### 3.3.1 fHSI data analysis

To extract the fluorescent dye composition at a given spatial location within the fHSI data cube, the data was processed as illustrated Figure 2B. A key aspect of the pre‐processing is background removal. When performing fluorescence imaging in reflectance mode, the excitation light is normally removed by band exclusion filters or by changing the light path using dichroic mirrors. For clinical applications, however, diffuse reflectance signals in the near‐infrared can give additional information related to blood oxygenation. We therefore exploited the spectral resolution of the HSI system to perform software background removal from the fluorescence data, such that for future *in vivo* applications the reflectance signal could also be used. We compared two methods: ‘brute force’ and orthogonal subspace projection (OSP). The conventional filter‐based reflectance removal was mimicked mathematically in software by ‘brute force’ exclusion of the spectral bands encompassing the peaks of the LED excitation. Orthogonal subspace projection (OSP) removes the principal components (PCs) of the ‘flat’ HSI cube (acquired before fluorescent dye administration) from the fHSI data cube to produce new endmembers 33, 34 (Figure 2E). OSP is a well established method of spectral background characterization in hyperspectral chemometrics 33. These software methods were compared to the introduction of crossed polarisers in hardware, which will remove the contribution of surface reflections, and the case without any reflectance removal.

The pre‐processed HSI cube was analysed using non‐negativity constrained least squares (NNLS) spectral unmixing based on data extracted from the ‘endmember’ HSI cubes (Figure 2B). The final output is an abundance map for each fluorescent dye and one for the background, where the intensity scale is the least squares (LS) score. Region of interest (ROI) data is extracted from these images for analysis.

#### 3.3.2 fHSI spectral unmixing performance

**Figure 3:**
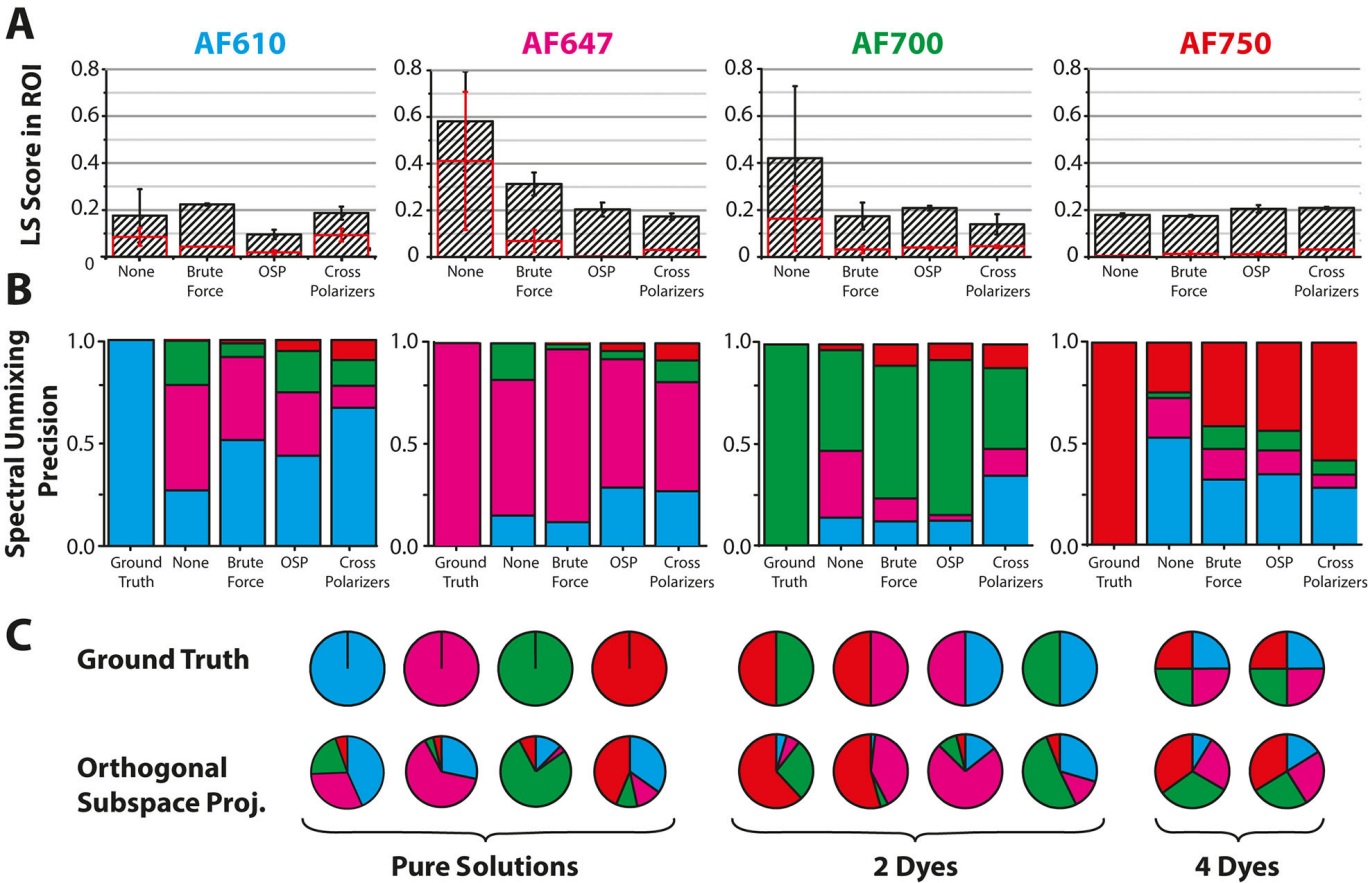
fHSI accurately identifies each fluorescent dye and is able to perform multiplexing. (A) Non‐negativity constrained least squares (NNLS) spectral unmixing was applied to well plates containing pure and mixed solutions of the different fluorescent dyes at 10 μM concentration, with and without background removal. The least squares (LS) scores from the dye abundance map within the expected well (black striped bars) and in a background well containing phosphate buffered saline (PBS) only (red bars) are compared. The error bars indicate the range over two dye and PBS wells in the same well plate. (B) Spectral unmixing precision (SUP) is the ratio of the LS score for the correct abundance map to the sum of scores recorded from the same well across all other fluorescent dye abundance maps (but not the background abundance map). For example, in the case of a well containing AF610, the LS scores are used to calculate the SUP as follows: SUP=AF610 / (AF610+AF647+AF700+AF750). The ground truth for each pure dye dilutions gives SUP=1. (C) Multiplexing capability is demonstrated using pie charts to show the SUP for pure dyes (average over two wells of 10 μM from dilution series) as well as 2 and 4 component mixtures.

We evaluated the impact of different background removal methods used in data pre‐processing on data quantification. ROIs of 65 pixels radius were manually placed over the wells in the well plate image to extract an average LS score over each well. Figure 3A shows the LS score recorded for the four fluorescent dyes at 10 μM; endmembers at 40 μM concentration were input to the NNLS so a LS score of 0.25 would be expected at 10 μM, with 0 for the background. In all cases, applying NNLS without background removal results in an incorrect LS score assignment to both the signal and background, with the exception of the signal from AF750 for which the fluorescence signal is well separated from the reflectance light. AF647 and AF700 show poorest performance without background removal, as expected based on the strong overlap of their emissions with the reflectance light. All background removal methods improve this to some extent, however, brute force and hardware cross polarisers removal both increase mis‐fitting to the background. The statistical method of OSP provided the optimal combination of accuracy in the signal and background, although consistently underestimates the LS score expected for the signal. In all cases, the AF610 dye is least well resolved, most likely due to the low detection sensitivity of the HSI camera in this spectral region (Figure 1D). Nonetheless, a quantitative response is observed based on the high R^2^ values for all linear fits to the LS scores of the whole dilution series across the range of dyes (AF610: *y*=0.0065*x*+0.027, *R* ^2^=0.69; AF647: *y*=0.021*x*−0.012, *R* ^2^=0.95; AF700: *y*=0.018*x*+0.02, *R* ^2^=0.94; AF750: *y*=0.019*x*−0.01, *R* ^2^=0.98).

In addition to the LS score, we defined the spectral unmixing precision (SUP): the ratio of the LS score for the correct abundance map to the sum of scores recorded from the same spatial location across all other fluorescent dye abundance maps. For each background removal method, the spectral unmixing precision is compared to the ground truth (Figure 3B). Again, the OSP method performs well across the board, qualitatively allowing a majority decision to be made regarding the dye or dye(s) present in a given location in the well plate. The F/# of the objective did not have any significant impact on these results (Supporting Information Figure S5). The results from 2 and 4 component mixtures are illustrated in Figure 3C compared to the ground truth. The fundamental limitation of fluorescent dye mixtures with broad overlapping emissions (not limited to our application) is ‘bleed over’. This refers to the fact that the emission of one fluorescent dye can immediately excite another in the same solution. For example, the area of overlap between the AF610 emission spectrum and the excitation spectra of the other dyes used may be up to 65 %, 48 % and 28 % for AF647, AF700 and AF750 respectively. Thus, while the primary constituents in each well can be identified in most cases, extraction of quantitative concentration data will be difficult. AF610 is consistently underestimated in mixtures, likely due to a combination of low camera sensitivity as noted above but also the overlap between AF610’s emission spectra and the reflectance light.

**Figure 4:**
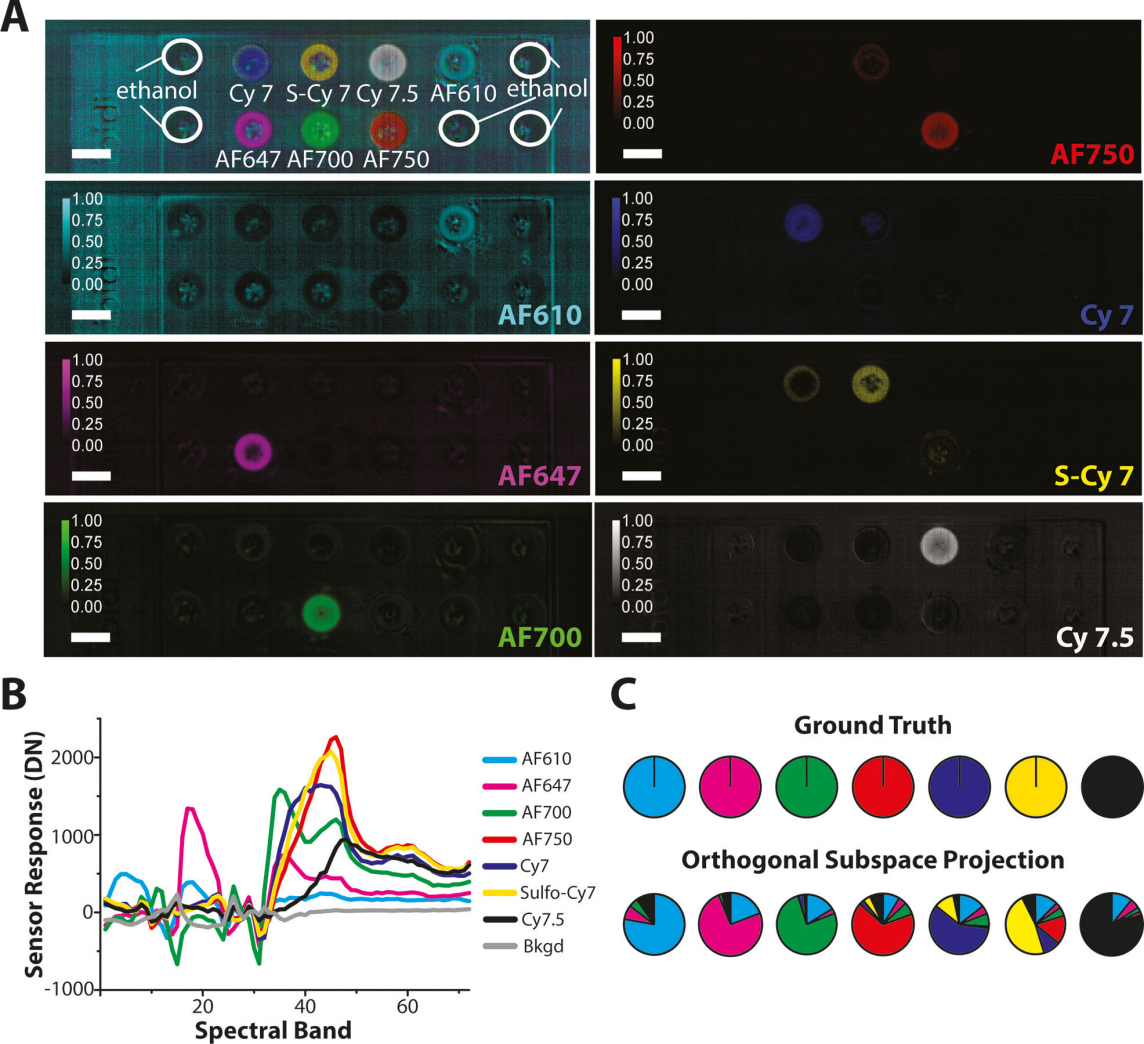
Up to 7 fluorescent contrast agents can be resolved in solution. 40 μM concentrations of AF610, 647, 700, 750, Cy7, S−Cy7 and Cy7.5 in ethanol were imaged on the same well plate and processed using OSP to produce abundance maps (A). The resulting endmembers (B) were used to identify the spectral unmixing precision (C), with only a limited degree of misfitting between AF750, Cy7, S−Cy7 and Cy7.5, which would be expected based on the similarity of their emission spectra.

To assess the response of the system in the case of more overlapping spectra, we introduced a well plate containing 3 additional fluorescent dyes (Cy7, S−Cy7 and Cy7.5) with significant spectral overlap with both the reflectance light and the existing AlexaFluor dyes. The abundance maps for each fluorescent dye is shown in Figure 4A. Based on measured endmembers (Figure 4B), all 7 dyes can be clearly resolved (Figure 4C). A limited degree of misfitting between AF750, Cy7, S−Cy7 and Cy7.5 was observed, which would be expected based on the similarity of their emission spectra.

To quantify the repeatability of the fHSI data, a well plate containing 4 wells of 30 μL of 40 μM dilutions of AF647 and 750 was imaged 8 consecutive times over a 35 minute time period. The coefficient of variation of the unmixed fluorescence signal (reflectance removal via OSP) with time was found not to exceed 6 % for any of the wells. Based on the low coefficient of variation, we can conclude that photobleaching of the dyes does not occur at the illumination levels and acquisition times used in this study. Well plates containing 30 μL of 40 μM dilutions of AF610, AF647, AF700 and AF750 were also imaged on four separate occasions over a 6 month period; the average coefficient of variation of the unmixed fluorescence signal from the dyes over the different imaging sessions was 16 %. The larger variations in the fluorescence signals is likely due to the different positioning of the well plate within the camera FOV between imaging sessions.

### 3.4 fHSI of multiple fluorescent dyes in tissue mimicking phantoms

**Figure 5:**
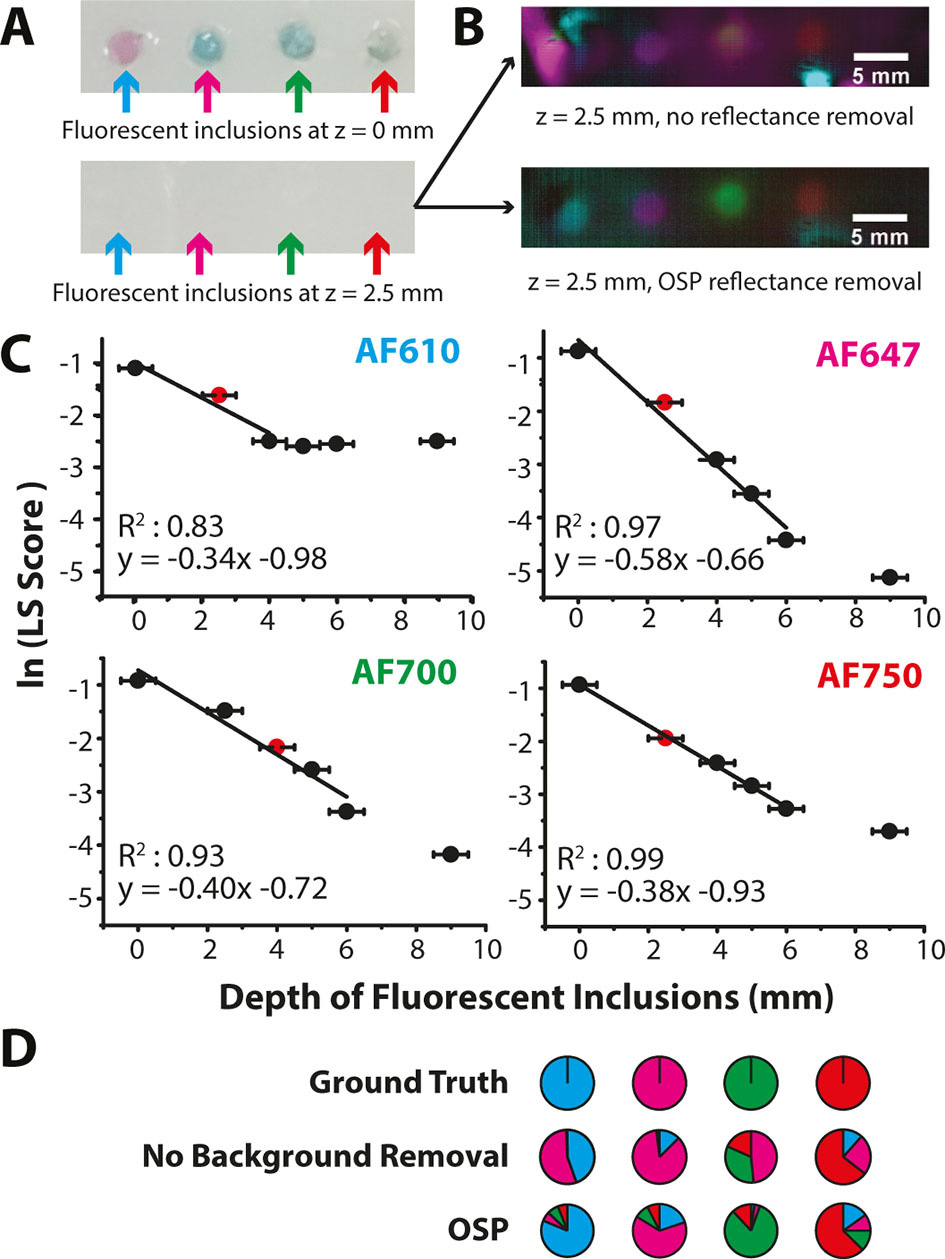
fHSI can detect four fluorescent dyes at depth in tissue mimicking phantoms. (A) Photograph of the phantom containing AlexaFluor dye inclusions before and after the addition of a 2.5 mm thick slab of tissue mimicking agarose gel. (B) Merged pseudocolor abundance maps for each dye with and without OSP background removal in pre‐processing. The maximum intensity value in each fluorescence abundance map is rescaled to a LS score of 1 for visualization. (C) The LS score of the fluorescent inclusions as a function of depth. The red data markers indicate the last data point with a weighted contrast‐to‐noise ratio (CNR) of 4 or higher. Error bars indicate the measurement uncertainty of the thickness of the agarose gel slabs. (D) A comparison of the spectral unmixing precisions show that whilst it is possible to identify the dyes based on a majority decision for the OSP pre‐processed data, this is not possible without background removal.

We next used tissue mimicking agarose phantoms to explore the spectral unmixing performance and depth sensitivity in a more realistic background. The original four AlexaFluor fluorescent dyes were individually placed in an intralipid phantom at a concentration of 40 μM, confined in the phantom using transparent plastic straws. Initially the phantom was imaged with the top of the straw exposed, then agarose gel slabs of up to 2.5 mm thickness were sequentially placed on top to gradually increase the depth (Figure 5A, B). Regions of interest of 50 pixel radius were manually placed over the inclusions or background regions to extract an average least squares (LS) score over each well. The weighted contrast to noise ratio (CNR) 35 was calculated taking the signal as the LS score correctly assigned to the fluorescent dye inclusion and the background as the LS score incorrectly assigned to the background region in the same fluorescence abundance map. CNR remained above 4 to depths of: AF610, 2.5 mm; AF647, 2.5 mm; AF700 4.0 mm and AF750, 2.5 mm (indicated in red in Figure 5C), while linearity persisted for up to a further 4 mm, with high R^2^ values of the linear fits. Spectral unmixing precision was also evaluated (Figure 5D), giving similar performance as in well plates. Full quantification data for LS score and SUP are shown in Supporting Information Figure S6.

### 3.5 fHSI of multiple fluorescent dyes *in vivo*

**Figure 6:**
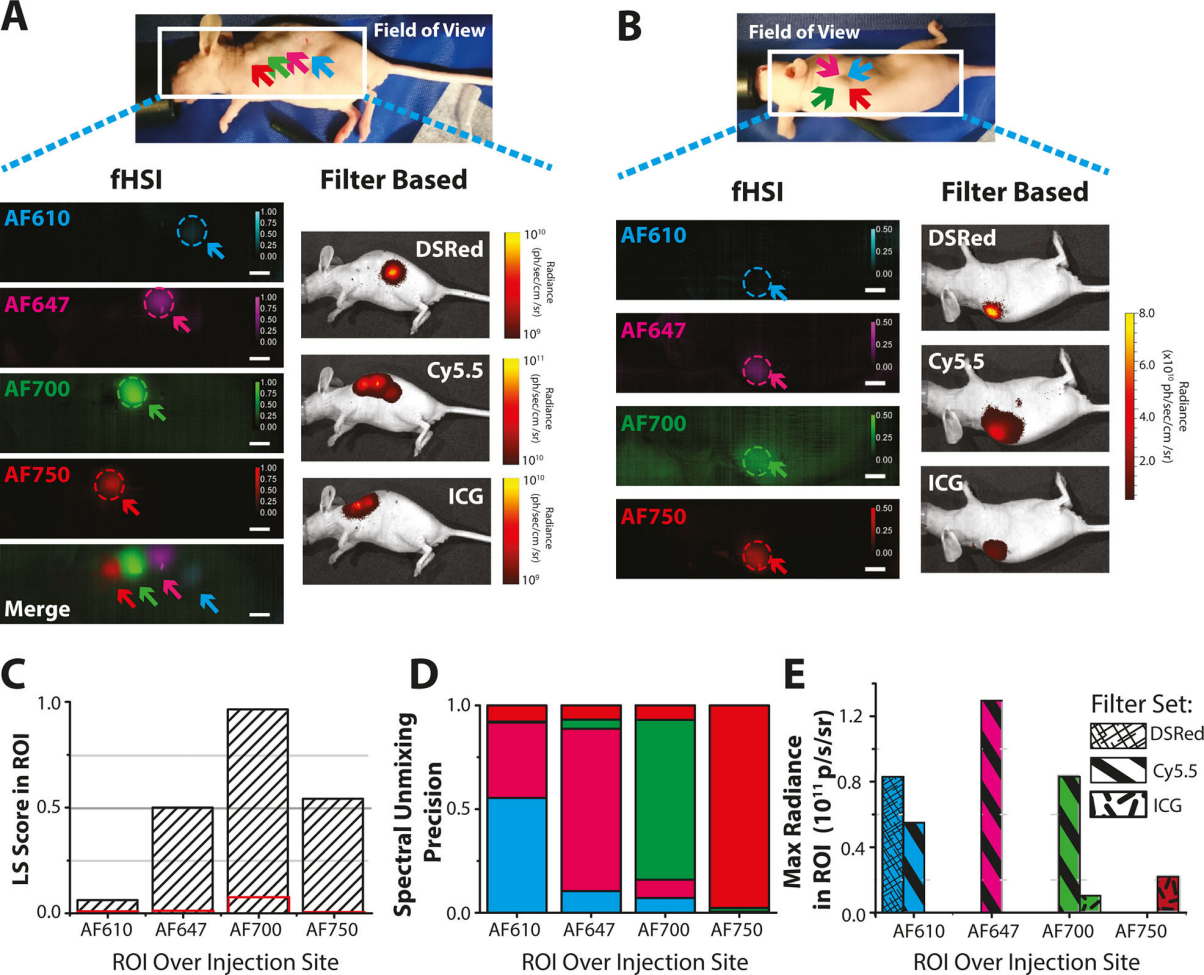
fHSI enables spectral resolution of four AlexaFluor dyes in living subjects. (A) Abundance maps and the pseudocolor images show clear resolution of separate injection sites (indicated by arrows) of each fluorescent dye for the fHSI. (B) Abundance maps from a mixed injection of all four dyes. Intensity scale bar indicates LS score in both cases, with spatial scale bar=5 mm. Data acquired using a filter based approach is shown for reference to the right. ROIs of 30 pixel radii were then placed over the separate subcutaneous injection site on the fHSI and filter‐based image data to extract average LS scores (C) (background in red defined as all pixels outside of the injection site ROIs), spectral unmixing precision (D) and from the filter‐based data, maximum radiance (E).

To assess the *in vivo* imaging performance of wide‐field fHSI, we administered subcutaneous injections of the four AlexaFluor fluorescent dyes (40 μM in a 1:1 solution of PBS and phenol red‐free matrigel) in the flank of a nude mouse maintained under anesthesia. Prior to imaging on the fHSI system, the successful injection and placement of the fluorescent dyes was verified on a filter based optical imaging instrument (IVIS200, Figure 6A, B). The locations of all four fluorescent dyes could be retrieved from their respective fHSI abundance maps (Figure 6A). In mixtures (Figure 6B), only three dyes can be observed clearly in the abundance maps, due to the challenges noted above with detection of AF610 in mixtures. The successful detection of the dyes on the fHSI system is reinforced by LS scores and SUP extracted from 30 pixel radii ROIs placed over the injection site of the separate subcutaneous dye injections (Figure 6C, D). As would be expected, the equivalent quantification from the filter‐based optical imaging instrument shows significant overlap between the recorded fluorescent dye emissions (Figure 6E), highlighting the previously established value of using fHSI approaches for *in vivo* imaging 10.

## 4 Discussion

Hyperspectral imaging (HSI) records both spatial and spectral information in a single data set. We hypothesized that the use of SRDAs could provide a compact and cost effective approach to acquisition of fHSI data, which would aid future clinical application of the approach. We first performed a technical characterization of the novel SRDA‐based HSI camera. Our HSI camera calibration explored the impact of illumination angle of incidence on the spectral response of the monolithically integrated filters 36, 37. In agreement with theory 31, we found that both optical throughput and FWHM decrease substantially as the F/# of the objective lens is increased. The coupling optics should therefore be carefully considered when integrating SRDAs into biomedical imaging systems. We found that an objective lens F/# of 1.65 offered a good compromise between sensitivity (high) and adequate spectral resolution (low) for the low light levels and broad spectra associated with the small molecule fluorescent dyes used here.

Integrating the camera into a reflectance‐based widefield imaging system provided the first proof‐of‐concept for fHSI using an SRDA. We exploited the spectral component of the HSI data cube for background removal using OSP. Our results demonstrated simultaneous imaging and unmixing of up to seven commonly used fluorescent dyes with highly overlapping emissions in pure solutions. When prepared in mixtures, fluorescent dyes naturally ‘bleed over’ i. e. the emission of one excites another, which affects any attempt to quantify concentration of highly overlapping dyes (independent of the imaging system used). We therefore focused on extracting quantitative data of four relatively well spectrally separated dyes. The presence of individual dyes at a given location could be identified based on majority decisions in our data: in superficial well plates; at depths of several millimetres in tissue mimicking phantoms; and *in vivo* in mice. We compared our *in vivo* to images from a traditional filterbased fluorescence system, which reinforces the advantage of increased spectral sampling for multiplexed fluorescence imaging.

Although our fHSI system shows promise, there remain some limitations to this study. Firstly, the system performance is limited by the low QE and dynamic range of the commercial CMOS image sensor used in the HSI camera. Future integration of the optical filters onto a scientific grade sensor could overcome the QE limitations. Dynamic range could be extended using a sensor with spatially programmable exposure times or through application specific filter design to exclude reflected excitation light. Secondly, while we were able to demonstrate quantitative imaging in solutions *in vitro*, achieving this *in vivo* is more challenging as sample topology and the optical properties of tissue can modulate the recorded fluorescence spectra and this must be carefully accounted for, especially when imaging at depth 17, 38. Thirdly, in this work, all data processing is performed offline but for many *in vivo* imaging applications, the abundance maps would need to be reconstructed in real time; transferring our data processing algorithms onto graphical processing units could achieve the required processing speeds. Finally, while pushbroom imaging is acceptable for static (or low temporal resolution) samples (e. g. for optical inspection of excised tissues after surgery), for video‐rate applications such as endoscopic and intra‐operative imaging, a pixel level deposition of filters is preferable, requiring a trade‐off between spectral and spatial resolution.

In summary, we have designed, characterized and applied a novel SRDA‐based HSI camera in a wide‐field reflectance‐based fluorescence imaging system. We demonstrated that our fHSI system shows promise for multiplexed fluorescence imaging in solutions *in vitro* and *in vivo* in mice. These data are highly encouraging, indicating that in addition to removing reflected light, fHSI combined with OSP could in the future enable diffuse reflectance and autofluorescence to be separately resolved from fluorescent dyes in clinical applications. Exploiting SRDAs, fHSI using targeted molecular imaging fluorescent contrast agents could find application in endoscopic cancer diagnosis and intra‐operative tumour resections, improving disease classification and margin identification respectively.

## Supporting information

Additional supporting information may be found in the online version of this article at the publisher's website.

## Acknowledgements

We thank Dale Waterhouse for scientific advice and comments on the manuscript, lab manager Fiona Morgan and the staff at BRU Cambridge Cancer Institute. We also thank Andy Lambrechts and the hyperspectral team at Imec for helpful discussions. This work was funded by CRUK (C47594/A16267, C14303/A17197), the EPSRC‐CRUK Cancer Imaging Centre in Cambridge and Manchester (grant no. C197/A16465) and the EU FP7 agreement FP7‐PEOPLE‐2013‐CIG‐630729. Additional funds were provided by the University of Cambridge MRC Confidence in Concept Award. ASL thanks the EPSRC, the George and Lillian Schiff Foundation and the Foundation Blanceflor for studentship funding.

## Supporting information

As a service to our authors and readers, this journal provides supporting information supplied by the authors. Such materials are peer reviewed and may be re‐organized for online delivery, but are not copy‐edited or typeset. Technical support issues arising from supporting information (other than missing files) should be addressed to the authors.

Supplementary

Supplementary
