# Supplementary material for: Fluorescence hyperspectral imaging (fHSI) using a spectrally resolved detector array
Source: J Biophotonics. 2017 May 9;10(6-7):840–53. doi: 10.1002/jbio.201600304 (PMC5953275; doi:10.1002/jbio.201600304)
Supplement: Supplementary file 1 — Supplementary [file JBIO-10-840-s001.pdf]

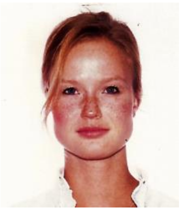

A. Siri Luthman graduated in 2013 with a first class MSci degree in Physics from Imperial College London UK. She is currently pursuing a PhD on *Hyperspectral Fluorescence Imaging for Biomedical Applications* in the Bohndiek group at the University of Cambridge.

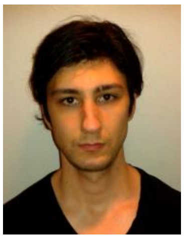

Sebastian F. Dumitru completed a first class undergraduate degree in 2016 at the University of Cambridge, UK. He is currently pursuing a Master's in Astrophysics at the University of Cambridge.

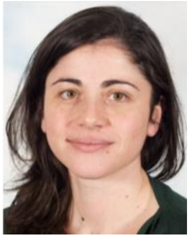

Isabel Quíros-Gonzalez obtained her MSc in Biology in 2005 and PhD in Biomedical Sciences in 2011 from the University of Oviedo, Spain. Isabel completed postdoctoral research at the Hospital Universitario Central de Asturias, Spain and subsequently as an EMBO fellow at the Wellcome Trust Sanger Institute, UK. She joined the Bohndiek group at the University of Cambridge, UK in 2015 and is interested in angiogenesis during cancer progression.

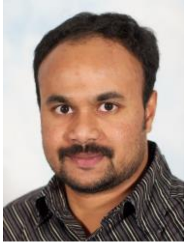

James Joseph obtained his PhD degree in 2013 from Nanyang Technological University, Singapore. Since 2014 he has been working as postdoctoral research associate in Bohndiek group at University of Cambridge. He develops and validates novel imaging and diagnostic approaches and instruments for biomedical applications.

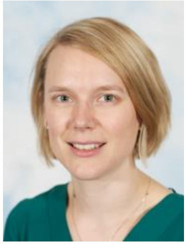

Sarah E. Bohndiek received her PhD from University College London, UK in Radiation Physics in 2008 and subsequently worked as a postdoctoral researcher at the University of Cambridge, UK and Stanford University, USA. Since 2013, she has established the VISIONLAB at the University of Cambridge, UK with a focus on clinical translation of new imaging and sensing technologies that can interrogate the tumour microenvironment.
