# Supplementary material for: Fluorescence hyperspectral imaging (fHSI) using a spectrally resolved detector array
Source: J Biophotonics. 2017 May 9;10(6-7):840–53. doi: 10.1002/jbio.201600304 (PMC5953275; doi:10.1002/jbio.201600304)

Supporting Information

© Copyright Wiley-VCH Verlag GmbH & Co. KGaA, 69451 Weinheim, 2016

Fluorescence hyperspectral imaging (fHSI) using a spectrally resolved detector array

Anna Siri Luthman, Sebastian Dumitru, Isabel Quiros-Gonzalez, James Joseph, and Sarah E Bohndiek*, This is an open access article under the terms of the Creative Commons Attribution License, which permits use, distribution and reproduction in any medium, provided the original work is properly cited.

Supporting Information for Journal of Biophotonics:

Fluorescence hyperspectral imaging (fHSI) using a spectrally resolved detector array

A Siri Luthman[1,2], Sebastian Dumitru[1], Isabel Quiros-Gonzalez[1,2], James Joseph[1,2] and Sarah E Bohndiek[1,2]

[1] Department of Physics, University of Cambridge, JJ Thomson Avenue, Cambridge, CB3 0HE, U.K.

[2] Cancer Research UK Cambridge Institute, University of Cambridge, Li Ka Shing Centre, Robinson Way, Cambridge, CB2 0RE, U.K.

***seb53@cam.ac.uk**

Supporting Methods

Details of Pre-Processing Data Corrections

Edge corrections were performed to account for light leakage between neighbouring spectral bands, which occur due to a slight mismatch with the filter edges and pixel positions, noted by the manufacturer. To correct for this, pixels at the edge of the spectral bands were replaced with the average pixel response of their adjacent pixels, representing a simple form of spatial denoising.

Spatial vignetting corrections are typically performed via flat fielding, however, this is not applicable for the current system due to the spectral selectivity of the HSI sensor and narrow spectral range of the illumination light, meaning that the LED light could only be used to correct a limited number of spectral bands on the sensor. Instead, an HSI data cube acquired with a broadband uniform LCD screen in the sample plane was instead pre-processed then normalized to extract a 'vignetting correction' mask image for each spectral band. The correction was applied by multiplying the reconstructed hyperspectral image of each spectral band with the inverse of the 'vignetting correction' mask image for that band.

Specular reflection removal was applied to data acquired from highly reflective plastic well plates without the crossed linear polarisers in place via ratiometric thresholding. The specular reflection removal is based on the assumption that specular reflections will skew the intensity ratio of the response within the two spectral bands (12 and 27) that respond maximally to the 660 and 732 nm LED colours. A ratiometric image was produced by

dividing the pre-processed image of spectral band 12 with spectral band 27. The mean and standard deviation of the pixels within the ratiometric images were calculated, and pixels outside the qualitatively determined Z-score value of 1.5 were rejected from subsequent analysis. Note that specular reflections are not present for the *in vivo* data and that specular reflection removal was therefore not applied to this data set.

Figure S1. Investigation of keystone factor. A regular grid pattern was overlaid on a Lambertian White Screen (SG3151-0; Sphere Optics) and illuminated with a broadband halogen source (OSL2 with OSL2B bulb; Thorlabs) to obtain image data in all spectral bands of the SRDA. (A) Illustrative image from spectral band 27. The mean length across the spectral bands analysed (1, 9, 18, 27, 36, 45, 54, and 72) against the known dimension of the grid pattern is shown in the horizontal (B) and vertical (C) directions. The high R^2=1 indicates that no keystone aberrations are present.

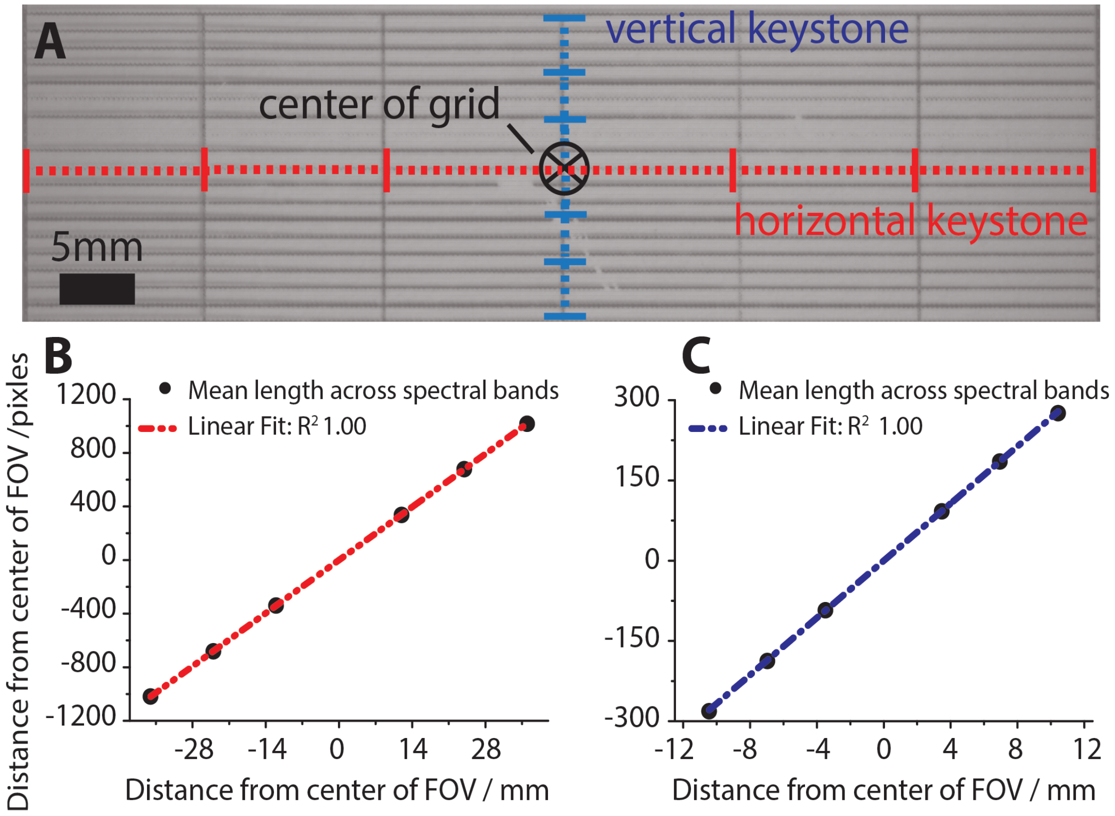

Figure S2. Investigation of spectral smile factor. Spectral smile was investigated by recording data from a Lambertian White Screen (SG3151-0; Sphere Optics) when illuminated with the 660 nm LEDs according to the protocol for imaging described in the main text. (A) Regions of interest (ROIs) were placed along the field of view of the HSI cube. (B) The extracted peak wavelength and full width at half maximum (FWHM) based on an objective F/# of 1.65 (see Figure S4) indicates that the spectral band responding to the reflected light changes from band 13 to band 11, corresponding to a change in wavelength of 6 nm. Given the FWHM of the band response is ~18 nm, the recorded spectral smile is negligible.

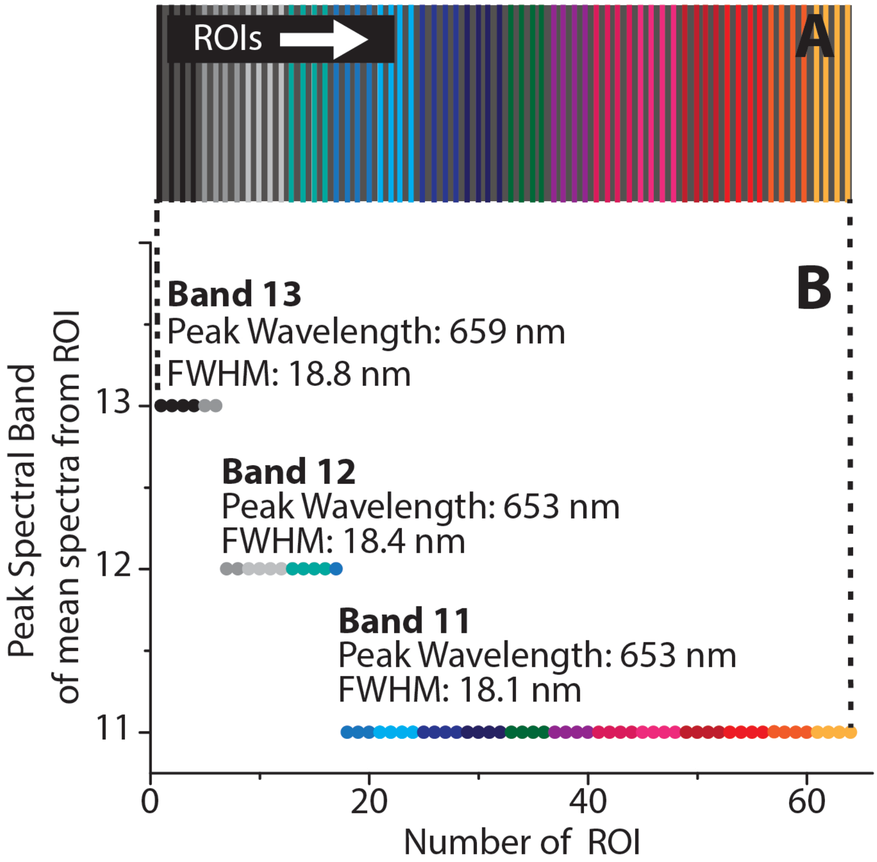

Figure S3. Edge Correction to account for light leakage between neighbouring spectral bands. (A) Representative example of the line artifact arising due to a slight mismatch between the filter edges and the CMOS sensor array pixel position. The image is shown for spectral band 11 from a HSI cube acquired using a uniform reflectance target illuminated with the 660nm LEDs. (B) shows the same image area after edge correction, showing that the line artifact has been removed. (C) The mean CMOS sensor array digital number output across the pixel rows for the non-edge corrected and corrected spectral band data. For this representative example the range of pixel values of pixel rows within the same spectral band decreased from 20% to 3% following edge correction.

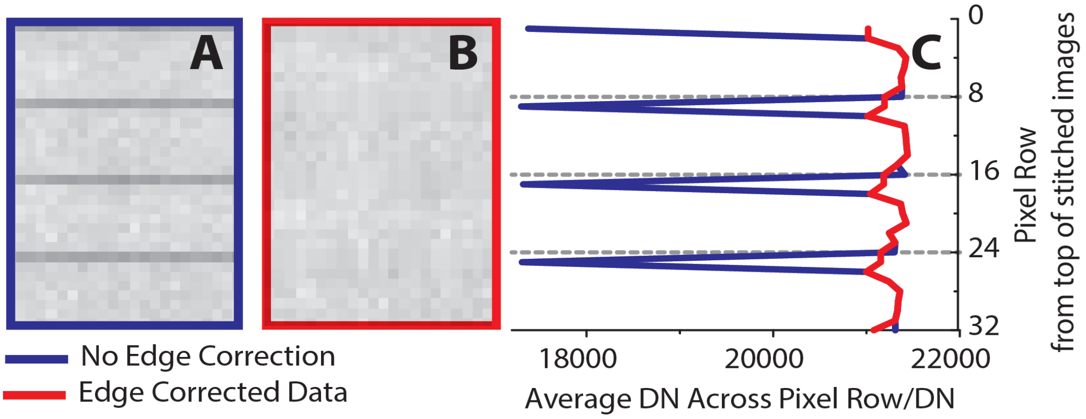

Figure S4. Response of the SRDA to collimated and diffuse light. (A) Calibrated quantum efficiency response of the spectral bands when illuminated with collimated light as shown in Figure 1 of the main manuscript. (B) Comparison of relative Spectral Response (SR) as a function of wavelength for different F/# settings of the variable wide-field objective used in this study.

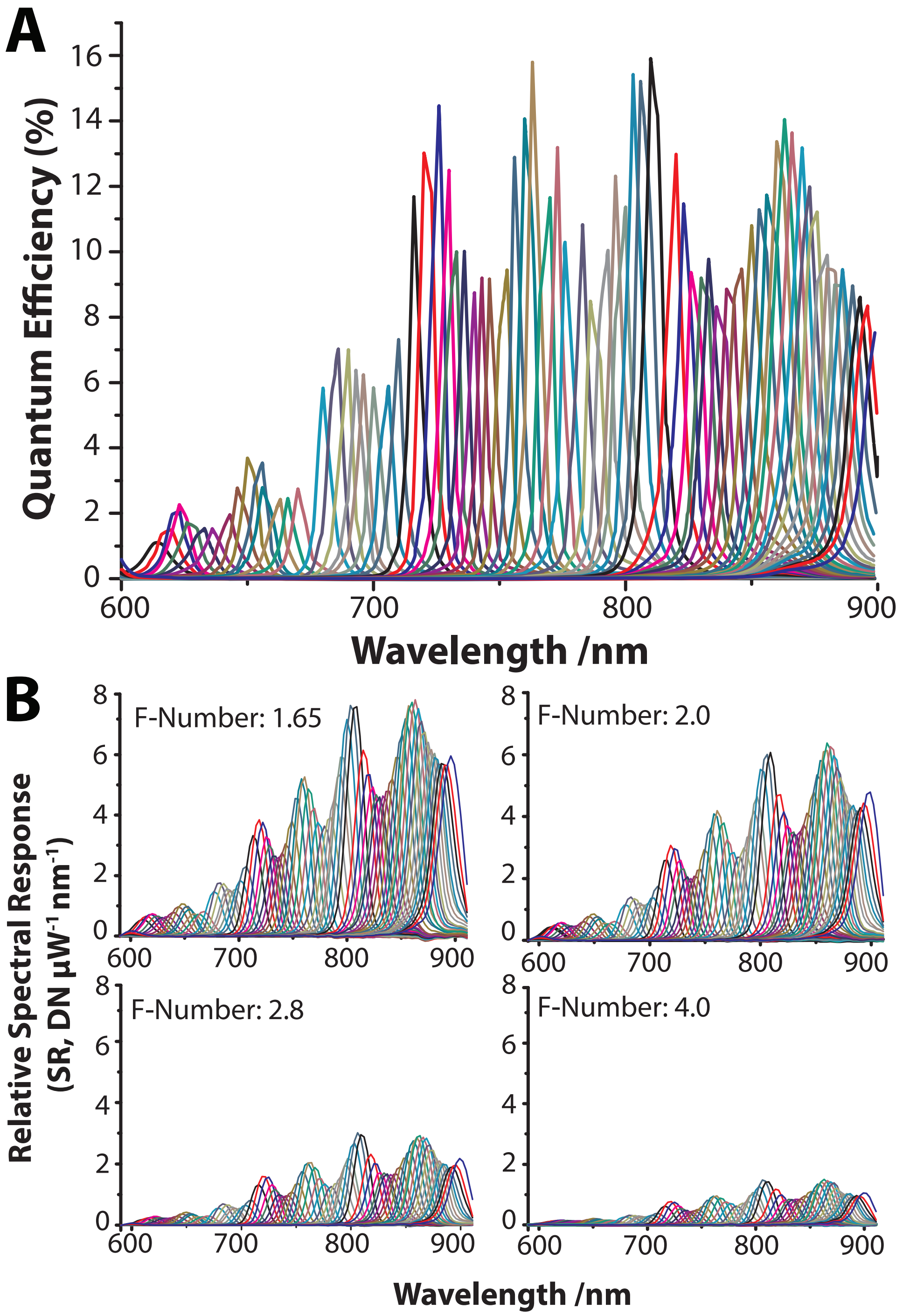

Figure S5. Impact of changing the objective F/# on the least squares (LS) score or spectral unmixing precision (SUP). (A) The LS score assigned to the ROIs placed over wells of a well plate (black, dye; red, background in a well containing phosphate buffered saline) imaged with the fHSI system for different objective lens F/#'s. For AF610, the combined optical throughput and sensitivity is too low to detect the dye emissions at a F/# of 4.0. The error bars indicate the range of the LS scores measured from two different wells containing dye and phosphate buffered saline respectively. (B) SUP of the dyes for increasing F/#'s. All data was pre-processed using orthogonal subspace projection.

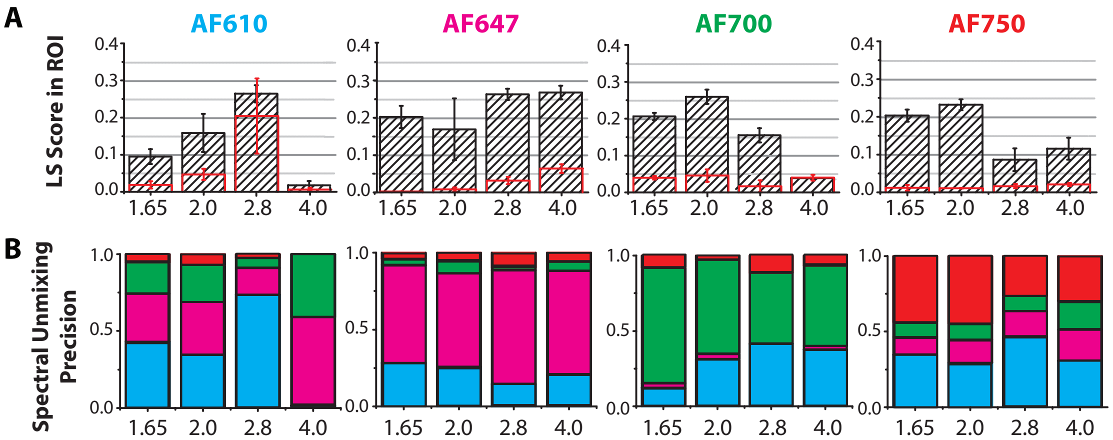

Figure S6. Quantification of LS score and SUP for tissue mimicking phantoms for different background removal methods in software. (A) LS score within ROIs placed over the dye inclusions (black striped bars) and over PBS inclusions (red bars) at 2.5 mm depth in tissue mimicking phantom. LS score is shown for no background removal (none), as well as brute force and OSP pre-processing. (B) SUP compared to ground truth.

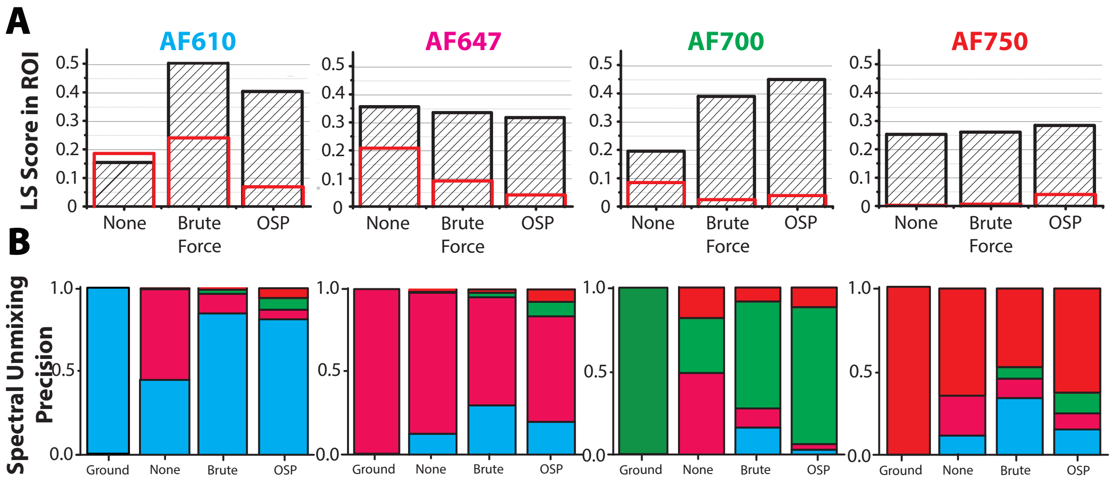

Supplement: Supplementary file 2 — Supplementary [file JBIO-10-840-s002.pdf]
